# Clinical implication of smoking among patients with schizophrenia at a Tertiary Institution in South East Nigeria

**DOI:** 10.4314/ahs.v18i1.14

**Published:** 2018-03

**Authors:** Chinyere Aguocha, Richard Uwakwe, Emmanuel Olose, Kennedy Amadi, Gabriel Onyeama, Chukwuma Duru

**Affiliations:** 1 Imo state university Teaching Hospital, Orlu, imo state, Nigeria, Department of Medicine; Imo state university , owerri, imo state, Nigeria, Department of Medicine; 2 Nnamdi Azikiwe University Teaching Hospital, Mental Health; 3 Department of Psychiatry, University Of Calabar, Cross Rivers, Nigeria; 4 University of Nigeria, Department of Psychological Medicine; 5 Imo State University Teaching Hospital, Orlu, Nigeria., Community Medicine

**Keywords:** Schizophrenia, smoking, psychopathology, disability, Nigeria

## Abstract

**Background:**

The chronic and debilitating nature of schizophrenia creates a disease with marked clinical and economic consequences. Smoking in schizophrenia appears to be associated with increased psychopathology and disability.

**Objective:**

The aim of this study was to determine if cigarette smoking in schizophrenia is associated with increased disability and psychopathology.

**Materials and methods:**

This was a cross-sectional descriptive study in which 367 out-patients with International Classification of Diseases (ICD) 10 diagnosis of schizophrenia were recruited. Socio-demographic questionnaire, Present State Examination (PSE) 10, Positive And Negative Syndrome Scale (PANSS) and World Health Organization Disability Assessment Schedule (WHODAS) were administered. Data was analyzed using a software package SPSS version 15.

**Results:**

There was no significant difference in the mean PANSS scores of smokers and non-smokers. Current smoking was associated with increased disability (F=5.39, p=0.02). Total PANSS score significantly predicted disability F(3,71=5.60, p=0.002, R2=0.19). There was no significant association between positive or negative symptoms and being a smoker or non-smoker.

**Conclusion:**

The results of this study revealed that smoking in Nigerian schizophrenia patients is associated with significant disability. Measures should be put in place to discourage cigarette smoking among Nigerian patients with schizophrenia.

## Introduction

Schizophrenia has been ranked as one of the ten most debilitating diseases worldwide and accounts for 1.1% of the total disability-adjusted life years and 2.8% of years lived with disability^1^,^2^. Studies that compared disability across mental disorders showed that schizophrenia ranked ahead of dementia, depression, obsessive compulsive disorder, alcoholism and bipolar affective disorder^3^,^4^,^5^. Schizophrenia leads to a breakdown in interpersonal and occupational achievement^6^. Psychopathology has been recognized as one of the most important factors in disability^7^. Disability in schizophrenia persists for years after symptom remission^8^. Disability affects social participation mainly and is associated with age of the patient and severity of psychotic symptoms^9^.

Nicotine is by far the most prevalent drug abused by schizophrenia patients and it increases mortality rate and financial burden incurred in the course of treatment^10^,^11^,^12^,^13^. The effect of smoking on the outcome of schizophrenia has been rather controversial^10^,^13^,^14^,^15^.

Understanding the effects of smoking on disability and psychopathology in schizophrenia would help develop appropriate evidence based biological and psychosocial interventions for these patients. No studies investigating the influence of cigarette smoking on the clinical expression of schizophrenia as measured via Positive And Negative Syndrome Scale (PANSS) and disability via World Health Organization Disability Assessment Schedule (WHODAS) have been reported in the Nigerian South East population. This article therefore explored the hypothesis that smoking leads to increased psychopathology and disability among patients with schizophrenia in South East Nigeria.

## Subjects and methods

This study was carried out at the Federal Neuropsychiatric Hospital located at Enugu, South Eastern region of Nigeria. The hospital provides services mainly to patients from the five states that make up the South Eastern part of Nigeria. A total of 367 patients with schizophrenia from the out-patient department of the hospital were studied.

This was a hospital based descriptive cross-sectional study. Three hundred and sixty seven patients with International Classification of Diseases (ICD 10) diagnosis of schizophrenia confirmed with Present State Examination 10 (PSE-10) and not too ill to undergo the interview were included in the study. Patients were excluded if they declined to take part and if by self report or from medical records it was found that they had any other important disorder besides schizophrenia.

WHODAS II 12 Item Version was used to measure disability. It is a measure of global disability. The participants were asked to rate the level of difficulty they experienced (none 1, mild 2, moderate 3, severe 4, extreme 5), taking into consideration the ease in which they performed a given activity. Simple sum scoring method was used to calculate total disability. The internal consistency and test-retest reliability of the overall WHODAS 2.0 is high. WHODAS II has been used previously in Nigeria^16^,^17^.

PANSS is a 30-item questionnaire that is used to assess patients' current mental state. The PANSS includes 30 items on 3 subscales: 7 items covering positive symptoms, 7 covering negative symptoms and 16 covering general psychopathology. Each item is scored on a seven-point item-specific Likert scale ranging from 1 to 7. Total Scores for the three scales were arrived at by summation of ratings across the component items. In addition, a composite scale was scored by subtracting negative from positive scores yielding a bipolar index (positive and negative), which was essentially a difference in score reflecting the degree of predominance of one syndrome in relation to the other^18^. Those who had equal scores in both scales were regarded as neither positive nor negative. PANSS has excellent internal consistency and inter-rater reliability. PANSS has been used previously in Nigeria^19^.

Patients who met the ICD 10 diagnosis of schizophrenia, made by a consultant psychiatrist or a senior trainee Psychiatrist (Resident Doctor) were selected. This diagnosis was confirmed using the schizophrenia schedule of PSE-10. Thereafter the socio-demographic questionnaire was administered to elicit information about age, gender, marital status, educational attainment employment status and age at onset of illness. For those who accepted ever smoking cigarettes, cigar or pipe in their life, PSE-10 smoking schedule was administered. The PSE-10 definition of “ever smoked” was adopted as the operational identification of “life time smoker”, whereas “current smoking” refered to “having smoked in the past four weeks”, irrespective of frequency or number of cigarettes^20^. Those who smoked 1–10 cigarettes per day were classified as light smokers while those who smoked more than 10 cigarettes everyday were classified as heavy smokers^21^,^22^. Early onset schizophrenia was classified as schizophrenia with onset before 18 years while late onset was above 18 years^23^.

We measured and quantified the severity of psychotic symptoms of schizophrenia using Positive and Negative Syndrome Scale (PANSS) in all the participants. The total PANSS Score was recorded. Finally World Health Organization Disability Assessment Schedule II 12 item version (WHODAS II) was administered to measure disability in the previous month in all the participants. The participants' WHODAS scores were dichotomized (yes/no) such that participants' with a total score of 12 were regarded as having no disability while scores above 12 indicated presence of disability^24^. Daily doses of neuroleptics were converted into chlorpromazine equivalents (CPZ-eqs) for ease of statistical analysis.

Data was cleaned manually and analyzed using Computer software (Statistical Package for the Social Sciences Version 15). Statistical testing was conducted at the 5% significance level and 95% confidence interval. Gender, educational level, age of the participants, age of onset of schizophrenia, marital status, religion and employment status were presented as simple frequencies and percentages. Chi-square was used to test association between symptoms of schizophrenia and smoking status. PANSS score, number of cigarettes smoked per day, severity of smoking and dosage of anti-psychotic medications were entered into a multivariable regression model to adjust for confounders and identify predicting associations between these variables and disability scores. Associations between the mean number of cigarettes smoked per day and psychopathology; and between the mean number of cigarettes smoked per day and disability were tested using t-test. A p-value <0.05 was considered significant.

This study was approved by the Ethics Committee of the Federal Neuropsychiatric Hospital Enugu. Voluntary informed consent was sought and obtained from all the participants before recruitment into the study.

## Results

Table 1 shows the characteristics of the participants. A total of 367 participants were included in the study. Most of the participants, 189 (51.5%) were females. Mean age of the participants was 34.1 ± 9.9 years. Mean age of onset of schizophrenia was 25.3 ± 6.5 years. The mean duration of schizophrenia illness was 8.76 ± 7.54. Most of the participants, 241 (65.7%) had never been married. Most of the participants had late onset schizophrenia (85.3%). One hundred and seventy six (48.0%) of the participants had a predominance of negative symptoms.

**Table 1:** Socio-demographic and clinical characteristics of participants

Variables	N	Percent (%)
**Gender**		
Female	189	51.5
Male	178	48.5
		
**Age (years)**		
10–29	134	36.5
30–49	196	53.4
50–69	37	10.1
Mean ± S.D	34.1 ± 9.9	
		
**Age of onset (years)**		
Early onset schizophrenia	54	14.7
Late onset schizophrenia	313	85.3
Mean ± S.D	25.3 ± 6.5	
		
**Duration of illness (years)**		
Mean ± S.D	8.76 ± 7.54	
		
**Marital status**		
Married	87	23.7
Never married	241	65.7
Divorced	32	8.7
Widowed	7	1.9
		
**Educational level**		
No formal education	16	4.4
Primary education	59	16.1
Secondary education	203	55.3
Tertiary education	89	24.2
		
**Religion**		
Christian	364	99.2
Islam	3	0.8
		
**Employment status**		
Employed	164	44.7
Unemployed	203	55.3
Smoking status		
Ever smoked	95	25.9
Currently smokes	75	20.4
		
**Severity of smoking**		
Light smokers	2	2.7
Heavy smokers	73	97.3
		
**Symptoms of schizophrenia**		
Predominance of positive symptoms	113	30.7
Predominance of negative symptoms	176	48.0
Equal dominance	78	21.3
Total PANSS Score	45.01±11.89	
		
**Disability**		
Yes	313	85.3
No	54	14.7
		
**Mean disability**	22.03 ± 8.35	

Part of Table 1 was adapted from Table 1 of ‘*Aguocha C.M., Aguocha J.K., Monday Igwe, Uwakwe R.U., Onyeama G.M. Prevalence And Correlates Of Cigarette Smoking Among Patients With Schizophrenia In South East Nigeria. Acta Psychiatrica Scandinavica 11 SEP 2014 | DOI: 10.1111/acps.12334*’

Table 2 shows that current smokers had a significantly higher mean WHODAS score of 24.0 ±8.1 compared to 21.5 ±8.3 for those that were not current smokers (F= 5.39, p= 0.02). The current smokers had a higher mean disability score (24.0 ±8.1) compared to the lifetime smokers (22.9 ±8.1). Among all the participants studied, the mean PANSS score was 45.01±11.89 with range 30–94. There was no significant difference in the mean PANSS scores of those who had never smoked and current and lifetime smokers. Multiple regression was used to predict disability from Total PANSS score, dosage of antipsychotics and number of cigarettes smoked per day. Total PANSS score significantly predicted disability β=0.35, F (3, 71 = 4.16, p=0.00, R2 = 0.19). There was a weak positive linear relationship between mean WHO-DAS Score and total PANSS Score (r=0.43, p= 0.0001) and between dosage of anti-psychotics prescribed and number of cigarettes smoked daily (r=0.27, p=0.009) among the current smokers.

**Table 2:** Mean WHODAS and PANSS scores among the participants.

Variable		Mean	Standard deviation	Test statistic	p-value

**WHODAS** **Ever smoked**	**Yes**	22.9	8.1	F= 1.52	p=0.22
	**No**	21.7	8.4		

**Currently smokes**					
	**Yes**	24.0	8.1	F= 5.39	p=0.02*
	**No**	21.5	8.3		

**PANSS** **Ever smoked**					
	**Yes**	44.2	9.5	F=0.6	p= 0.44
	**No**	45.3	12.6		

**Currently smokes**					
	**Yes**	44.8	10.0	F=0.04	p=0.85
	**No**	45.1	12.3		

F=One Way ANOVA

Table 3 showed greater rate of smoking among those with predominantly negative symptoms. There was no significant association between number of cigarettes smoked per day, disability scores (t=−1.04, p=0.30) and PANSS composite scores (t=−0.73, p=0.47).

**Table 3:** Association between smoking, disability and clinical psychopathology

Variable	Frequency	Percent	Stat	p-value
**Lifetime smoking**				
	**Yes (%)**	**No (%)**		
Predominance of Negative symptoms	50 (52.6)	126 (46.3)	X^2^ =5.72	p=0.06
Predominance of Positive symptoms	33 (34.8)	80 (29.4)	**d.f=2**	
Equal dominance	12 (12.6)	66 (24.3)		
**Current smoking**				
Predominance of Negative symptoms	41 (54.7)	135 (46.2)	X^2^ =4.88	p=0.09
Predominance of Positive symptoms	25 (33.3)	88 (30.2)	**d.f=2**	
Equal dominance	9 (12.0)	69 (23.6)		
**Mean No. of cigarettes smoked per day and disability**				
**Disability**	**N (%)**	**Mean No. of** **cigarettes smoked** **per day**	**Stat**	**p-value**
Yes	71 (94.7)	3.90 ± 3.62	t= −1.04	p=0.30
No	4 (5.3)	2.00 ± 0.82		
**Mean No. of cigarettes smoked perday and psychopathology**				
	**N (%)**	**Mean No. of** **cigarettes smoked** **per day**	**Stat**	**p-value**
Predominance of Positive symptoms	25	3.56 ± 3.26	t= −0.73	p=0.47
Predominance of Negative symptoms	41	4.24 ± 4.23		

## Discussion

Research regarding the clinical characteristics of cigarette smokers with schizophrenia has produced mixed results, which may be attributed to methodological and sampling differences^25^. Reduced rate of smoking in these patients compared to those in developed countries, differing antipsychotic medications, dosage of anti-psychotics and male to female ratio can account for this. A high rate of heavy smoking (97.3%) was found among the participants in this study. It was higher than 52% previously reported^26^. This difference can be attributed to variations in the cut off scores for heavy smoking^26^. Countries, especially developed countries with higher rate of smoking may tend to use a higher cut off^20^.

Most of the participants (48%) had a predominance of negative symptoms. This is higher than 25% and 15.8% reported in some other studies^27^,^28^. This may be due to differences in sampling methods, help seeking behavior, duration of untreated psychosis, long schizophrenia illness and effect of medications leading to an increase in negative symptoms^27^,^28^. Long duration of untreated psychosis found in developing countries, has been recorded among patients with predominantly negative symptoms^28^. Current smoking was demonstrated to be significantly associated with disability. This is consistent with what has been found in some other studies^29^,^30^. This has been attributed to smoking being a marker for a more severe schizophrenic illness^25^,^31^.

There was no significant association between smoking status and psychopathology. This is consistent with what has been previously reported^32^. In fact, contrary to what was expected, smokers had lesser total clinical psychopathology scores compared to non-smokers. This may be due to smoking inducing relaxation and pleasurable feelings and reducing anxiety, anger and depression^33^. These effects may have greater importance among patients with schizophrenia because their other sources of pleasure and satisfaction are likely diminished^33^. This supports what has been reported previously that there is little evidence to support that tobacco use worsens the symptoms of schizophrenia^14^,^34^.

Smoking status was not significantly associated with predominance of positive or negative symptoms. This agrees with reports of no statistically significant differences between schizophrenia smokers and non-smokers with regard to the severity of negative symptoms^30^,^35^,^36^. Some other studies have reported that patients with more severe negative symptoms tend to smoke more to alleviate the symptoms of schizophrenia and that more severe negative symptoms were independently associated with current smoking^37^,^38^,^39^. Reduced dopamine neurotransmission associated with chronic substance may be the cause of increased negative symptoms among these patients^10^. Clinical psychopathology was independently associated with disability among current smokers. This supports studies that have reported that though modest, increased psychopathology was associated with increased disability^40^. It also agrees with reports of total PANSS and Brief Psychiatric Rating Scale (BPRS) scores having a significant positive correlation with disability^13^,^41^.

## Limitation

The study was a cross-sectional non-population representative study with small number of participants, it included only out-patients with schizophrenia, so its results cannot easily be generalized to the entire population. Secondly, the estimation of cigarette smoking habits was by self-report. A hundred percent reliability cannot be guaranteed. Assessing nicotine dependence with more specific methods, such as pack- year smoking history and Fagerstrom test for nicotine dependence is required in further studies.

## Conclusion

Smoking in Nigerian schizophrenia patients is associated with significant disability and psychopathology. It calls for urgent measures to be implemented to discourage smoking among Nigerian patients with schizophrenia.
